# Supplementary material for: Effects of Caffeine Intake on Self-Administered Sleeping Quality and Wearable Monitoring of Sleep in a Cohort of Young Healthy Adults
Source: Nutrients. 2025 Apr 29;17(9):1503. doi: 10.3390/nu17091503 (PMC12073117; doi:10.3390/nu17091503)
Supplement: Supplementary file 1 [file nutrients-17-01503-s001.zip › nutrients-3612624-supplementary.pdf]

Outcome Variables

- Self-assessed questionnaires containing validated questions on:

  - Demographics (sex, age)
  - Pittsburgh Sleep Quality Index (PSQI) (12)
  - Problems falling asleep (Never, Rarely, Sometimes, Often, Always)
  - Waking up at night (Never, Rarely, Sometimes, Often, Always)
  - Problems feeling rested (Never, Rarely, Sometimes, Often, Always)
  - Intake of sleeping medications (Never, Less than once a week, >= once a week)
  - Problems staying awake during daytime (Never, Less than once a week, >= once a week)
  - Problems managing daily routines due to insufficient sleep (No problems, Rarely, Sometimes, Often)
  - Sleep quality
  - Daily bedtime (baseline: average over the last 4 weeks; follow-up: average over the last week)
  - Sleeping hours (baseline: average over the last 4 weeks; follow-up: average over the last week)

- Smartwatch tracking (Withings ScanWatch)

  - Overall duration of sleep
  - Duration of light sleep
  - Duration of deep sleep
  - Time to fall asleep: indicating the duration from lying down to falling asleep
  - Time to get up: indicating the duration from awakening to getting up
  - Heart rate
